# Dental Colleges

**Published:** 1851-10

**Authors:** 


Dental Colleges.—We have the Republican Banner, published at Nashville, Tenn., containing some three well written articles on the necessity of Dental Colleges and the practicability of such an institution in Nashville. The institution which went into operation, last winter, at Lexington, Kentucky, has made no provision as yet for a course this coming winter, and we are told, by one or two of the faculty, that they expect to open it hereafter in Louisville. We have no doubt but the interest of the profession and public will lutimately demand several such institutions. We shall always hail with pleasure every such effort for the advancement of Dental science.

We fear, however, that as yet the profession is not prepared to sustain so many dental schools. We believe that as yet there is but the one East—the Baltimore School—and if they cannot sustain more than one, we of the West should hardly be expected to keep in operation three.

We, however, believe that an additional school, in New York or Philadelphia, properly organized and sustained by the profession, would be of service to the schools already organized, and very materially assist in breaking up that suicidal course pursued by so many practitioners. There is not an argument which can be advanced against Dental Colleges which is not equally applicable to medical. We commend to the candid consideration of the profession Dr. E. Townsend's article on Dental Colleges, in the American Journal; also an article, in the October number of the Journal, by Dr. Ballard, on Dental Education

We hope to give one or both of these articles in a future number of the Register. Believing that there is only one side to the question of dental education, we shall do all that we can to advance it.

